# Predicting response to a community‐based educational workshop on incontinence among community‐dwelling older women: Post hoc analysis of the CACTUS‐D trial

**DOI:** 10.1002/nau.24614

**Published:** 2021-02-05

**Authors:** Xavier Fritel, Eleanor van den Heuvel, Adrian Wagg, Stéphanie Ragot, Cara Tannenbaum

**Affiliations:** ^1^ INSERM CIC 1402, CHU de Poitiers Université de Poitiers Poitiers France; ^2^ Université de Montréal Montréal Québec Canada; ^3^ Brunel Institute for Ageing Studies Brunel University Uxbridge UK; ^4^ University of Alberta Edmonton Alberta Canada

**Keywords:** ageing, educational workshop, urinary incontinence

## Abstract

**Aims:**

Our goal was to identify which women participating in an educational workshop on incontinence were most likely to benefit from it.

**Methods:**

We included women aged 65 or older, living in the community, and not treated for incontinence despite reporting urinary leakage at least twice a week. The workshop's aims were to change beliefs about accepting incontinence as a normal part of ageing, explain that incontinence is not irreversible, and that solutions exist. We performed structured interviews at 6 and 12 months to assess impressions of improvement (PGI‐I) and changes in both continence (ICIQ‐FLUTS) and quality of life (I‐QOL).

**Results:**

The analysis included 392 women, 39% aged 80 or older and 57% with daily urinary incontinence. Twelve months after the workshop, 16% of women were “much better” (PGI‐I); factors associated with impression of improvement were refusal to believe that incontinence is part of normal ageing at baseline and improvement of urinary symptoms. The median improvement was 4 points on the ICIQ‐FLUTS and 8 on the I‐QOL. Factors associated with a clinically significant improvement in urinary symptoms were more severe baseline urinary incontinence, obesity, and starting Kegel exercises. Factors associated with a clinically significant improvement in quality of life were a poor urinary quality of life at baseline and an age younger than 81 years.

**Conclusions:**

A short, inexpensive and nonmedical intervention can change the mind‐set and behavior of older women with incontinence who are not seeking care. A clinically significant improvement is possible even in women with severe symptoms.

## 1 INTRODUCTION

Urinary incontinence (UI) is common among older women and has been associated with loss of autonomy and impaired quality of life.^1^, ^2^, ^3^, ^4^ Despite its negative sequelae, the vast majority of incontinent women do not seek remedial treatment.^5^ We conducted the Continence across Continents to Upend Stigma and Dependency (CACTUS‐D) randomized trial to assess whether a community‐based health education program delivered to untreated women with incontinence could improve urinary symptoms and quality of life by raising awareness about incontinence and the potential for treatment and encouraging women to take up self‐management.^6^, ^7^

One of the most frequent reasons for the lack of care‐seeking for incontinence is the belief among women and their healthcare providers that no real solution exists to treat it.^6^, ^8^, ^9^ Beliefs are a primary driver of behavior, including adherence to treatments for incontinence, as shown with successful uptake of pelvic floor training.^10^ We showed in the CACTUS‐D trial that an educational program on incontinence that changes beliefs and promotes self‐management and care‐seeking leads to improved symptoms among older untreated women.^11^ Among the women included in the trial, those who participated in the incontinence self‐management workshop reported more frequent impressions of improvement (15% “very much better” or “much better”) a year later, as well as a significant improvement in their quality of life compared with the women exposed to a healthy ageing control workshop.^11^

One of the difficulties encountered in implementing the CACTUS‐D trial was the reluctance of women from community organizations to attend incontinence workshops, possibly because of the taboo and stigmatizing nature of this condition. Our goal in this predictive analysis was therefore to identify which women were most likely to benefit from the incontinence self‐management workshop to be able to target future participation in community workshops. We anticipated that women with the most severe symptoms could be expected to improve most frequently and most substantially, although it also seemed possible that women suffering from the most severe incontinence could be the least likely to benefit, either because they had already tried the measures taught during the workshop without success in the past, or because their effect would be insufficient.

## 2 MATERIAL AND METHODS

Our target population was women aged 65 years or older, living in the community, not treated for UI despite reporting urinary leakage at least twice a week and allocated to the incontinence self‐management workshop. The design of the CACTUS‐D trial has been described in detail^7^, ^11^: it was an open‐label, multinational cluster randomized controlled trial conducted across Quebec, Western Canada, the UK, and France that compared the effects of attendance at an incontinence self‐management workshop to that at a control healthy ageing workshop.

### 2.1 Intervention

The incontinence self‐management workshop led by a research assistant lasted approximately 1 h, delivered to groups of 1 to 10 women. The aim of the workshop was to change erroneous beliefs about accepting incontinence as a normal part of ageing, to explain that UI is not inevitable and irreversible, and that solutions to incontinence exist, such as performing pelvic floor contraction exercises regardless of age. The incontinence self‐management workshops in French or English can be accessed at this link: https://www.deprescribingnetwork.ca/cactus-trial or https://www.youtube.com/watch?v=xocZKg7GI5M&feature=youtu.be.

### 2.2 Baseline measures taken at the workshop

The severity of UI symptoms was assessed by the validated 12‐item International Consultation on Incontinence Questionnaire‐Female Lower Urinary Tract Symptoms (ICIQ‐FLUTS) symptom questionnaire. The higher the ICIQ‐FLUTS score (between 0 and 48), the more severe the urinary symptoms.^12^ The quality of life related to UI was assessed by the incontinence quality of life (I‐QOL) validated 22‐item questionnaire.^13^ The higher the I‐QOL score (from 0 to 100), the better the quality of life.

We asked participating women whether they practised Kegel exercises and if they thought UI was part of normal ageing; the late introduction of the second question explains why these answers are not available for the first women included. We also collected information about the women's demographic characteristics and lifestyle habits (age, education level, smoking, coffee or tea consumption, and body mass index), comorbidities (self‐reported health status, depression, high blood pressure, diabetes, and falls), pelvic floor health (duration of UI, fecal incontinence, and constipation).

### 2.3 Outcomes

We conducted structured telephone follow‐up interviews with participants at 6 and 12 months after the workshop to assess their impressions of their improvement, incontinence symptoms, and quality of life.

The women reported their impressions of improvement with the 7‐point Patient Global Impression of Improvement or PGI‐I Likert scale: “very much better,” “much better,” “a little bit better,” “no change,” “a little bit worse,” “much worse,” or “very much worse.” To explore changes in their beliefs or behaviors, we asked women again at follow‐up if they thought UI was part of normal ageing and whether they practised Kegel exercises.

Improvement in UI and quality of life scores were defined by a favorable change between baseline and 12 months on the ICIQ‐FLUTS and I‐QOL questionnaires equal to or greater than a predefined minimum clinical difference (MCID). When the improvement did not reach the MCID, the participant was not considered improved. The MCID for I‐QOL was estimated at 4.74. This threshold was calculated a priori by comparing the gain in the I‐QOL score of participants who considered themselves “much better” or “very much better” with participants who reported poorer quality of life or no improvement in a previous study.^6^, ^11^, ^14^ The MCID for the ICIQ‐FLUTS was calculated by the same method: women who considered themselves “much better” or “very much better” after the workshop had decreased their average score by −6.69 while women who rated themselves “a little bit better,” with “no change,” “a little bit worse,” “much worse,” or “very much worse” had a decrease in their score of −3.50. This allowed us to estimate the MCID for the ICIQ‐FLUTS at −3.19. In the absence of the 12‐month follow‐up data, we used, when available, the 6‐month follow‐up data (Figure S1 flow‐chart). Scores for women with missing values were calculated by replacing any missing answers by the mean of the answers given in the domain. When the number of answers given in one domain of a questionnaire was insufficient (response to fewer than a third of the questions asked in the domain), the score was not calculated and the participant was excluded from the analysis.

### 2.4 Analysis

We used three logistic regression models to compare the women who improved and those who did not improve among those attending the incontinence self‐management workshop: one model with PGI‐I, one with urinary symptoms (ICIQ‐FLUTS), and the last with urinary quality of life (I‐QOL) as the outcome measure. Predictors of improvement included demographic characteristics and lifestyle habits, comorbidities, pelvic floor health, and changes observed during follow‐up (change in ICIQ‐FLUTS and I‐QOL scores for the regression about improvement defined by the PGI‐I, Kegel exercises, and beliefs about UI). Variables eligible for maximal predictive models were the variables with *p* < .10 in the univariate analysis; we performed a backward manual procedure to eliminate nonsignificant variables; the final model contained all significant variables.

We assessed the c‐index of each model, which made it possible to measure its predictive performance (the closer the result is to 1, the greater the model's predictive ability, and the closer it is to 0.50, the less informative it is). We looked at the correlation between the ICIQ‐FLUTS changes and the I‐QOL changes (Spearman's correlation test) and determined how many women showed improvement (MCID or greater) in their ICIQ‐FLUTS scores, I‐QOL scores, and both.

We examined the characteristics of women who did not change their beliefs about UI as a normal part of ageing and those who started pelvic floor exercises.

We conducted a sensitivity analysis excluding women reporting very good quality of life (*n* = 49 with an I‐QoL score > 95.26) at baseline, because a ceiling effect prevented them from reaching the MCID.

## 3 RESULTS

The randomization procedure allocated 552 incontinent women aged 65 years or older and reporting at least two UI episodes weekly for which they received no care to the intervention group; they participated in the incontinence self‐management workshop. Among them, 392 women (71%) were followed‐up for 12 months after the workshop, completed questionnaires (flow‐chart, Figure S1), and constitute our analysis population.

### 3.1 Baseline characteristics

Of the women in our sample at baseline, 39.0% were 80 years of age or older and 99.5% had a mean of 8 comorbidities. Daily incontinence was reported by 57.1%: “once a day” for 28.0% and “several times a day” for 29.1%. Incontinence type was classified as stress urinary incontinence for 49 women (12.5%), urgency urinary incontinence for 109 (27.8%), mixed incontinence for 188 (48.0%), and other or nonclassifiable incontinence types for 46 (11.7%). Frequencies and leakage circumstances are reported in Table S1. At baseline, 191 women (48.7%) thought urinary incontinence was normal for their age and 205 (52.3%) did not perform Kegel exercises.

The women differed slightly according to their recruitment center: women in Québec reported depression less frequently, while women in Alberta were more likely to report constipation, smoking, or high blood pressure. Those in the UK reported the mildest urinary symptoms, and women included in France were the youngest, had the lowest level of education, poorest self‐reported health, and lowest likelihood of performing Kegel exercises (additional Table S2).

### 3.2 Prevalence of improvement

**Figure 1:**
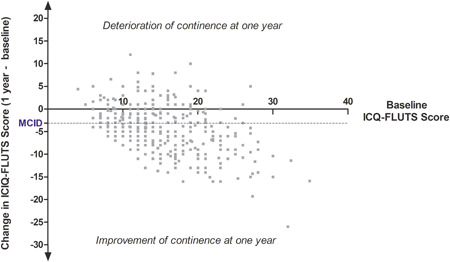
Changes in ICIQ‐FLUTS scores between baseline and follow‐up (dotted line for the minimal clinically important difference, MCID, −3.19). Negative change reflects improvement in continence. ICIQ‐FLUTS, International Consultation on Incontinence Questionnaire‐Female Lower Urinary Tract Symptoms; MCID, minimum clinical difference

**Figure 2:**
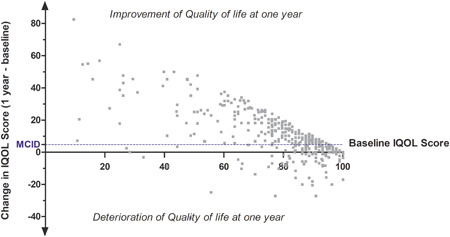
Changes in I‐QOL scores between baseline and follow‐up (dotted line for the minimal clinically important difference, MCID, +4.74). Positive change reflects improvement in Quality of Life (QoL)

At follow‐up, the median change in the ICIQ‐FLUTS score was an improvement of 4.0 points (interquartile range [IQR] 1.0–7.0; Figure 1). For the I‐QOL, the median improvement in score was 8.0 points (IQR 2.3–17.9; Figure 2). Improvement in the ICIQ‐FLUTS score was moderately correlated with that in the I‐QOL (Spearman coefficient −0.41, *p* < .001). A significant PGI‐I improvement (“very much better” or “much better”) was reported by 61 women (15.6%). A significant improvement reaching MCID or greater in the urinary symptom score and the urinary quality of life score was observed among 216 (55.1%) and 242 (61.7%) women, respectively (Figures 1 and 2); and 156 (39.5%) women reported a clinically significant improvement in both symptoms and urinary quality of life.

### 3.3 Predictors of patient‐reported global improvement

Compared with participants who persisted in their belief that incontinence was a normal part of ageing (*n* = 141, 51% of women responding at baseline and follow‐up), the women who changed this belief (*n* = 50, 18%) or did not initially believe that incontinence was a normal part of ageing (*n* = 83, 30%) were more likely to improve (Table 1). Self‐reported improvement of urinary symptoms on the ICIQ‐FLUTS at follow‐up was also associated with significant change on the PGI‐I.

**Table 1:** Factors associated with the Patient's Global Impression of Improvement at follow‐up (“much better” or “very much better” according to PGI‐I)

			Improved	Maximal model	Final model
Characteristics		*n*, or mean (*SD*)	%, or mean (*SD*)	Adjusted OR (95% CI)	Adjusted OR (95% CI)
Center	Montréal, Québec	191	15.7	1	1
	Edmonton, Alberta	46	10.9	0.78 (0.22–2.70)	0.75 (0.23–2.46)
	Uxbridge, UK	85	15.3	0.96 (0.32–2.90)	0.96 (0.32–2.85)
	Poitiers, France	70	18.6	1.84 (0.61–5.54)	1.34 (0.47–3.78)
Believe that incontinence is a normal part of ageing at baseline/follow‐up^a^	No/no or yes	83	16.9	2.83 (1.10–7.25)	2.91 (1.15–7.36)
	Yes/no	50	20.0	3.35 (1.18–9.55)	3.45 (1.27–9.40)
	Yes/yes	141	6.4	1	1
Self‐reported health status	Excellent or very good	123	21.1	1	
	Good	187	12.8	0.45 (0.18–1.12)	
	Fair or poor	81	13.6	0.61 (0.20–1.82)	
Depression	Yes	88	9.1	0.36 (0.10–1.34)	
	No	304	17.4	1	
Improvement in urinary symptoms (ICIQ‐FLUTS score)^b^		−4.2 (5.0)	−6.6 (5.8)	1.10 (1.00–1.20)	1.12 (1.04–1.21)
Improvement in HRQoL (I‐QoL score)^b^		+10.4 (14.7)	+14.8 (13.3)	1.02 (0.99–1.05)	
c‐index				0.75	0.72

*Note*: The following variables were not significant (*p* > .10) in the univariate analysis and were not introduced into the maximal multivariate model: smoking, education level, high blood pressure, diabetes, constipation, incontinence duration, fecal incontinence, daily coffee or tea, age, BMI, I‐QoL at baseline, ICIQ‐FLUTS at baseline, Kegel exercises at baseline/follow‐up, and falls. The final model retained only significant variables after backward elimination.

Abbreviations: BMI, body mass index; CI, confidence interval; ICIQ‐FLUTS, International Consultation on Incontinence Questionnaire‐Female Lower Urinary Tract Symptoms; I‐QoL, incontinence quality of life; OR, odds ratio; PGI‐I, Patient Global Impression of Improvement.

a Response unavailable for the first women included.

b OR was calculated for an improvement of the score of 1 point, that is, a variation between baseline and follow‐up equal to −1 for ICIQ‐FLUTS and + 1 for I‐QoL.

Of the 191 women who believed that incontinence was part of normal ageing at baseline, those who did not change their minds after the workshop (yes/yes in Table 1) were older (79 vs. 74 years of age) than those who modified their perceptions (yes/no in Table 1). Among the women who changed their beliefs, 33% initiated Kegel exercises compared to only 25% among women who did not (difference not significant).

### 3.4 Predictors of urinary symptom improvement

Baseline factors associated with a clinically significant improvement (MCID or greater) in urinary symptoms included greater UI severity and obesity (Table 2).

**Table 2:** Factors associated with significant improvement in urinary symptoms (change in ICIQ‐FLUTS score ≤ −3.15). Maximal and final multivariate logistic regression

			Improved	Maximal model	Final model
Characteristics		*N*	%	Adjusted OR (95% CI)	Adjusted OR (95% CI)
Center	Montréal, Québec	191	56.5	1	1
	Edmonton, Alberta	46	63.0	1.49 (0.72–3.06)	1.43 (0.70–2.93)
	Uxbridge, UK	85	55.3	1.59 (0.87–2.91)	1.58 (0.87–2.88)
	Poitiers, France	70	45.7	0.73 (0.40–1.35)	0.74 (0.40–1.35)
BMI (kg/m²)	−18.5	8	75.0	2.54 (0.44–14.59)	2.36 (0.42–13.18)
	18.5–24	142	52.8	1	1
	25–29	138	48.6	0.79 (0.48–1.31)	0.77 (0.47–1.26)
	+30	94	68.1	1.92 (1.06–3.48)	1.79 (1.01–3.20)
Urinary symptoms (ICIQ‐FLUTS^a^) at baseline	1st tertile: ≤ 12.0	146	39.0	1	1
	2nd tertile: 12.1–17.0	135	57.8	2.65 (1.51–4.64)	2.31 (1.36–3.92)
	3rd tertile: > 17.0	111	73.0	5.35 (2.73–10.49)	4.36 (2.41–7.86)
HRQoL (I‐QoL) at baseline^b^	3rd tertile: ≥ 88.6	137	51.1	1	
	2nd tertile: 76.2–88.5	129	51.9	0.72 (0.41–1.25)	
	1st tertile: < 76.2	126	62.7	0.65 (0.34–1.21)	
Kegel exercises at baseline/follow‐up	Yes/no or yes	184	51.1	1	1
	No/yes	113	66.4	1.96 (1.15–3.31)	1.93 (1.15–3.26)
	No/no	92	48.9	1.19 (0.68–2.07)	1.17 (0.67–2.04)
c‐index				0.70	0.70

*Note*: The following variables were not significant (*p* > .10) in the univariate analysis and were not introduced into the maximal multivariate model: smoking, education level, health situation, high blood pressure, diabetes, falls, belief that incontinence is a normal part of ageing at baseline/follow‐up, constipation, incontinence duration, fecal incontinence, daily coffee or tea every day, age, and depression. The final model contained only significant variables after backward elimination.

Abbreviations: BMI, body mass index; CI, confidence interval; ICIQ‐FLUTS, International Consultation on Incontinence Questionnaire‐Female Lower Urinary Tract Symptoms; I‐QoL, incontinence quality of life; OR, odds ratio; PGI‐I, Patient Global Impression of Improvement.

^a^ The higher the ICIQ‐FLUTS score (between 0 and 48), the more severe the urinary symptoms.

^b^ The higher the I‐QOL score (between 0 and 100), the better the quality of life.

Among the 205 women who did not perform Kegel exercises at baseline, the 113 (55%) who started them (no/yes in Table 2) reported an improvement in urinary symptoms after the workshop; they were younger (76 vs. 80) than those who did not (no/no in Table 2). The rate of women starting Kegel exercises differed significantly between the centers: 63% in the UK, 62% in Québec, versus 50% in Alberta and only 36% in France. We did not find an association between starting Kegel exercises and a change in belief about UI.

### 3.5 Predictors of improvement in quality of life

Baseline factors associated with a clinically significant improvement (MCID or greater) in urinary quality of life were a poor baseline urinary quality of life and an age younger than 80 years. Women recruited from the Poitiers center (France) were least likely to report an improvement in their urinary quality of life (Table 3). The findings did not change when the 49 women with an excellent baseline urinary quality of life (I‐QoL score > 95.26) were excluded (sensitivity analysis).

**Table 3:** Factors associated with significant improvement in urinary quality of life (change in I‐QoL score ≥ + 4.74). Maximal and final multivariate logistic regressions

			Improved	Maximal model	Final Model
Characteristics		*N*	%	Adjusted OR (95% CI)	Adjusted OR (95% CI)
Center	Montréal, Québec	191	67.0	1	1
	Edmonton, Alberta	46	71.7	1.33 (0.58–3.03)	1.32 (0.58–2.99)
	Uxbridge, UK	85	60.0	1.08 (0.57–2.05)	0.99 (0.53–1.85)
	Poitiers, France	70	42.9	0.31 (0.16–0.60)	0.32 (0.16–0.61)
Age	1st tertile: ≤ 72	125	64.8	1	1
	2nd tertile: 73–80	128	67.2	1.02 (0.55–1.88)	1.04 (0.57–1.91)
	3rd tertile: ≥ 81	139	54.0	0.52 (0.28–0.94)	0.53 (0.29–0.95)
Urinary symptoms (ICIQ‐FLUTS) at baseline^a^	1st tertile: ≤ 12.0	146	46.6	1	
	2nd tertile: 12.1–17.0	135	68.1	1.44 (0.79–2.62)
	3rd tertile: > 17.0	111	73.9	1.16 (0.58–2.33)
HRQoL (I‐QoL) at baseline^b^	3rd tertile: ≥ 88.6	137	31.4	1	1
	2nd tertile: 76.2–88.5	129	69.8	5.58 (3.15–9.91)	6.02 (3.45–10.51)
	1st tertile: < 76.2	126	86.5	13.17 (6.43–26.99)	14.18 (7.42–27.09)
c‐index				0.81	0.80

*Note*: The following variables were not significant (*p* > .10) in the univariate analysis and were not introduced into the maximal multivariate model: smoking, education level, health situation, high blood pressure, diabetes, falls, belief that incontinence is a normal part of ageing at baseline/follow‐up, constipation, incontinence duration, fecal incontinence, daily coffee or tea every day, BMI, depression, Kegel exercises at baseline/follow‐up. The final model contained only significant variables after backward elimination.

Abbreviations: BMI, body mass index; CI, confidence interval; ICIQ‐FLUTS, International Consultation on Incontinence Questionnaire‐Female Lower Urinary Tract Symptoms; I‐QoL, incontinence quality of life; OR, odds ratio; PGI‐I, Patient Global Impression of Improvement.

^a^ The higher the ICIQ‐FLUTS score (between 0 and 48), the more severe the urinary symptoms.

^b^ The higher the I‐QOL score (between 0 and 100), the better the quality of life.

## 4 DISCUSSION

Our analysis shows that beliefs about incontinence predicted a positive response to community‐based continence promotion among older women, but the consistency of the observation depended on the outcome measure used. These beliefs were strongly related to self‐reported global impressions of improvement, but not to changes in symptom or quality of life scales. Obese women, those aged 80 years or younger, with severe urinary symptoms at baseline, or an initial lower quality of life appeared more likely to benefit from the intervention, as assessed by urinary symptom and quality of life outcome measures.

One of the limitations of our work is that the data were exclusively self‐reported and have not been clinically verified. The study design was intended to include untreated women living in the community who had not sought care for incontinence, which meant that no clinical evaluations were available. There are significant disparities in how women, compared with healthcare professionals, rate improvement of incontinence, but arguably the patient's global perception is most relevant.^15^ We do not know to what extent the women understood and implemented the information provided by the workshop, or which part of the intervention they found most useful. It is nonetheless interesting to note that two thirds of the women who started doing Kegel exercises after the workshop reported an improvement in their urinary symptoms. Similarly, women who changed their beliefs about incontinence being a normal part of ageing were 3 times more likely to say they felt much better.

One of the strengths of this study is its multinational and multicultural character. This made it possible to investigate the effect of the education program across different populations and thus to be able to study the prognostic factors for the workshop's success. Because we did not choose the participating women, we believe that they were similar to the target population of women attending the community organizations.

Among the characteristics of the incontinence self‐management workshop evaluated in the CACTUS‐D study, we must take into account the lack of individual adaptation and clinical expertize. There is an essential difference between an educational workshop and medical care. The facilitator in charge of the workshop was not a clinician and did not know the severity of the symptoms, the history, or the expectations of the women present.

It was interesting to observe a positive effect on urinary symptoms in women who reported the most severe symptoms and a positive effect on quality of life among those with the lowest urinary quality of life. These results reinforce the rationale of the message delivered during the workshop: that urinary incontinence is not part of normal ageing and it is always possible to do something. One of the obstacles to improvement in our analysis was believing otherwise,^16^ and it was more difficult to change these beliefs about urinary incontinence among the oldest women.

Despite this obstacle, our work shows that it was possible to change the beliefs of a quarter of older women who thought that urinary incontinence was normal and to change the behavior of more than half of those who did not do Kegel exercises. Some might consider the workshop effect modest, comparing unfavorably with other treatments. This effect was, however, obtained without any medical expertise or intervention. A short, inexpensive, and nonmedical intervention was able to change the mind‐set and behavior of older women with incontinence who had not sought care. Our analysis shows that a significant improvement of continence is possible even in cases with severe symptoms.

It seems therefore appropriate to offer the workshop to older women living in the community, even those with severe incontinence and/or obesity. Some modifications of the workshop, to tailor it more specifically to supporting behavior change (e.g., ongoing support and discussion of barriers) may enhance its effect. They remain to be tested.

## 5 CONCLUSIONS

Our continence promotion program can change the behavior of older women with incontinence who do not seek care and can induce a significant improvement despite severe symptoms.

## CONFLICT OF INTERESTS

Adrian Wagg reports grants from Canadian Institutes of Health Research during the conduct of the study; Xavier Fritel, Eleanor van den Heuvel, Stéphanie Ragot, and Cara Tannenbaum have nothing to disclose.

## Supporting information

Supporting information.

## Data Availability

Data available on request from the authors.
