# Supplementary material for: Predicting response to a community‐based educational workshop on incontinence among community‐dwelling older women: Post hoc analysis of the CACTUS‐D trial
Source: Neurourol Urodyn. 2021 Feb 5;40(2):705–13. doi: 10.1002/nau.24614 (PMC8247852; doi:10.1002/nau.24614)
Supplement: Supplementary file 1 — Supporting information. [file NAU-40-705-s001.docx]

**Supporting information:**

Table S1. Frequency and leakage circumstances (ICIQ-FLUTS) at baseline (N=392)

| Frequency | Leakage circumstances, n (%) | | | |
| --- | --- | --- | --- | --- |
|  | “before you can get to the toilet” | “when you are physically active, exert yourself, cough or sneeze” | “for no obvious reason and without feeling that you want to go” | “when you are asleep” |
| “never” | 18 (4.6%) | 55 (14.1%) | 171 (43.6%) | 252 (64.3%) |
| “occasionally” | 70 (17.9%) | 98 (25.0%) | 98 (25.0%) | 76 (19.4%) |
| “sometimes” | 221 (56.4%) | 144 (36.7%) | 92 (23.5%) | 40 (10.2%) |
| “most of the time” | 61 (15.6%) | 80 (20.4%) | 18 (4.6%) | 14 (3.6%) |
| “all of the time” | 15 (3.8%) | 15 (3.8%) | 8 (2.1%) | 6 (1.5%) |

Table S2: Characteristics of participating women at baseline (N=392)

|  | | |  | | Overall population N= 392 | Montréal, Québec N= 191 | Edmonton, Alberta N= 46 | Uxbridge, UK N= 85 | Poitiers, France N= 70 |
| --- | --- | --- | --- | --- | --- | --- | --- | --- | --- |
| At baseline | | |  | | n (%) or mean (±SD) | | | | |
| Smoking* | | |  | | 14 (3.6) | 8 (4.2) | 3 (6.5) | 2 (2.3) | 1 (1.4) |
| One cup of tea/coffee or more per day | | | | | 352 (89.8) | 176 (92.1) | 42 (91.3) | 76 (89.4) | 58 (82.8) |
| Age (years) | | |  | | 77.2 (±7.8) | 78.0 (±7.7) | 79.3(±7.6) | 75.8 (±7.6) | 75.6 (±8.2) |
| BMI (kg/m²) | | |  | | 27.1 (±5.3) | 26.9 (±5.2) | 27.5(±5.2) | 27.0 (±4.5) | 27.2 (±6.5) |
| Education level: more than 12 years* | | | | | 181 (46.2) | 96 (50.3) | 23 (50.0) | 43 (50.6) | 19 (27.1) |
| Self-reported health status* | excellent or very good good fair or poor | | | | 123 (31.5) 187 (47.8) 81 (20.7) | 64 (33.7) 91 (47.9) 35 (18.4) | 19 (41.3) 17 (37.0) 10 (21.7) | 31 (36.5) 37 (43.5) 17 (20.0) | 9 (12.9) 42 (60.0) 19 (27.1) |
| Hypertension* | | |  | | 214 (54.6) | 113 (59.2) | 31 (67.4) | 44 (51.8) | 26 (37.1) |
| Diabetes | | |  | | 68 (17.3) | 43 (22.5) | 7 (15.2) | 11 (12.9) | 7 (10.0) |
| Depression* | | |  | | 88 (22.4) | 25 (13.1) | 18 (39.1) | 20 (23.5) | 25 (35.7) |
| Experienced a fall in previous years | | | |  | 158 (40.3) | 76 (39.8) | 20 (43.5) | 33 (38.8) | 29 (41.4) |
| Believe that incontinence is a normal part of ageing | | | | | 269 (68.6) | 134 (70.2) | 35 (76.1) | 59 (69.4) | 41 (58.6) |
| Performing pelvic floor muscle exercises (Kegel)* | | | | | 185 (47.2) | 94 (49.2) | 26 (56.5) | 40 (47.1) | 25 (35.7) |
| ICIQ-FLUTS score (0-48)* | | |  | | 15.1 (±5.5) | 16.4 (±5.7) | 15.6(±4.6) | 12.6 (±5.1) | 14.3 (±4.5) |
| I-QOL score (0-100) | | |  | | 77.9 (±18.9) | 76.0 (±19.8) | 74.9 (±20.2) | 80.7 (±18.5) | 81.6 (±14.9) |
| Incontinence duration | | Less than one year 1 to 5 years More than 5 years | | | 53 (13.5) 160 (40.8) 171 (4.6) | 26 (13.6) 73 (38.2) 90 (47.1) | 9 (19.6) 19 (41.3) 17 (37.0) | 8 (9.4) 37 (43.5) 37 (43.5) | 10 (14.3) 31 (44.3) 27 (38.6) |
| Constipation* | | |  | | 127 (32.4) | 48 (25.1) | 22 (47.8) | 33 (38.8) | 24 (34.3) |
| Leak stool | | |  | | 74 (18.9) | 35 (18.3) | 9 (19.6) | 16 (18.8) | 14 (20.0) |

* significant difference between centres

**Figure 1**: Flow-chart

No

N= 9

**Each questionnaire (PGI-I, I-QOL, ICIQ-FLUTS) completed at follow-up?**

6-month visit missing

N= 8

1-year visit missing

N= 16

**Yes: Analysis population**

**N= 392**

Lost to follow-up
N= 151

**1 year follow-up**

**N= 385**

**6-month follow-up**

**N= 393**

**552 women included
in the intervention group**
